# Y chromosomal evidence on the origin of northern Thai people

**DOI:** 10.1371/journal.pone.0181935

**Published:** 2017-07-24

**Authors:** Andrea Brunelli, Jatupol Kampuansai, Mark Seielstad, Khemika Lomthaisong, Daoroong Kangwanpong, Silvia Ghirotto, Wibhu Kutanan

**Affiliations:** 1 Department of Life Science and Biotechnology, University of Ferrara, Ferrara, Italy; 2 Department of Biology, Faculty of Science, Chiang Mai University, Chiang Mai, Thailand; 3 Center of Excellence in Bioresources for Agriculture, Industry and Medicine, Chiang Mai University, Chiang Mai, Thailand; 4 Department of Laboratory Medicine & Institute for Human Genetics, University of California San Francisco, San Francisco, California, United States of America; 5 Forensic Science Program, Faculty of Science, Khon Kaen University, Khon Kaen, Thailand; 6 Department of Biology, Faculty of Science, Khon Kaen University, Khon Kaen, Thailand; University of Florence, ITALY

## Abstract

The Khon Mueang represent the major group of people present in today’s northern Thailand. While linguistic and genetic data seem to support a shared ancestry between Khon Mueang and other Tai-Kadai speaking people, the possibility of an admixed origin with contribution from local Mon-Khmer population could not be ruled out. Previous studies conducted on northern Thai people did not provide a definitive answer and, in addition, have largely overlooked the distribution of paternal lineages in the area. In this work we aim to provide a comprehensive analysis of Y paternal lineages in northern Thailand and to explicitly model the origin of the Khon Mueang population. We obtained and analysed new Y chromosomal haplogroup data from more than 500 northern Thai individuals including Khon Mueang, Mon-Khmer and Tai-Kadai. We also explicitly simulated different demographic scenarios, developed to explain the Khon Mueang origin, employing an ABC simulation framework on both mitochondrial and Y microsatellites data. Our results highlighted a similar haplogroup composition of Khon Mueang and Tai-Kadai populations in northern Thailand, with shared high frequencies of haplogroups O-PK4, O-M117 and O-M111. Our ABC simulations also favoured a model in which the ancestors of modern Khon Mueang originated recently after a split from the other Tai-Kadai populations. Our different analyses concluded that the ancestors of Khon Mueang are likely to have originated from the same source of the other Tai-Kadai groups in southern China, with subsequent admixture events involving native Mon-Khmer speakers restricted to some specific populations.

## Introduction

The area of northern Thailand situated in proximity to southern China, northern Myanmar and northern Laos hosts several ethnicities who can be linguistically classified in four groups: Austroasiatic, Tai-Kadai (TK), Hmong-Mien and Sino-Tibetan. The languages belonging to the Austroasiatic subfamily Mon-Khmer (MK) are spoken today by populations, e.g. Lawa (LW) and Mon (MO), historically and archaeologically recorded as the native inhabitants of this area before the arrival of Tai-Kadai people form southern China 2,000 years ago [1–3]. Other ethnicities, such as the Hmong-Mien (e.g. Hmong) and different Sino-Tibetan groups (e.g. Karen), migrated from nearby countries to the mountainous areas of northern and western Thailand no more than 200 years ago [4–5]. In addition, other recent migrations from southern China and/or northern Myanmar are recorded as involving several Tai-Kadai groups such as Lue (LU), Khuen (KH), Yong (YO) and Shan (SH) [6].

Linguistic similarity between populations has often been used to disentangle patterns of relationship, under the assumption that a common language implies a common origin [7,8]. However, genetic similarities between different populations are often more complex than expected from linguistic data due to the effect of processes such as drift and migration [9]. The mountainous area of northern Thailand consists of several river plains surrounded by mountains, which continue from the Shan Hills in bordering Myanmar to Laos. In this region, dissimilar geographic areas are often occupied by different ethnolinguistic groups. The hill tribes, such as LW and SH speaking groups, currently live on the mountain where mobility is quite limited while, on the other hand, populations such as MO and different TK speaking groups inhabit the well-connected lowland regions. Geography, acting in addition to cultural/linguistic isolation, might have been an influential factor in determining the divergence by inbreeding of these populations [10]. Due to the combined effect of these different processes, northern Thailand is an extremely interesting site for studies on human population genetics.

Among the multi-ethnic groups in northern Thailand, the Khon Mueang (KM) are the most represented, with a total number of individuals reaching 6 million [11]. KM is the name with which local northern Thai people, possibly the Yuan (YU), call themselves, and refers more to a past social and political category rather than a distinct population [12,13]. Linguistically speaking, the KM’s language is similar to that of the YU, which is classified as belonging to the TK family.

It is widely accepted that genetic markers can be efficiently used to reconstruct past populations’ history and interactions [14–20]. Three types of genetic markers are commonly used to reconstruct past population processes, differing in the modality of inheritance: the maternally inherited mitochondrial DNA (mtDNA), paternally inherited Y chromosome, and the autosomal and X chromosomes that are inherited by both parents. Even after the rise of whole-genome techniques, uniparental markers continue to be widely used in population genetics. Due to their specific patterns of inheritance, the information provided by the Y-chromosome and by the mitochondrial DNA (mtDNA) allows for in depth analyses of sex-specific patterns of population history and demography. These data can be used to locate asymmetric contributions of male and female individuals to a migration, an event of admixture and generally to the pattern of gene-flow in a certain area.

A previous study, based on comparisons among autosomal Short Tandem Repeat (STR) loci, suggested an admixed origin for KM, with a higher contribution from the TK than from the MK groups [21]. On the other hand, a coalescent modelling using mtDNA genome data indicated southern China as the most probable origin of the KM, without admixture with LW groups in northern Thailand [22]. Y chromosomal data of KM and of their linguistic and geographic neighbours in northern Thailand have been reported, but they have been limited to STR markers [10,23]. Here, we investigated newly generated data of single nucleotide polymorphism (SNP) on Y chromosome along with previously published Y-STRs and mtDNA data. We used a combination of classical statistical analyses and model based simulations to shed light on the past population dynamics linked to the origin of KM of northern Thailand.

## Material and methods

### Y binary markers and assembled datasets

A sample of 519 males belonging to 24 populations from northern Thailand was subdivided into three groups: Khon Mueang (KM), Mon-Khmer (MK) and Tai-Kadai (TK) (Table 1). The DNA samples were obtained from our previous studies with written informed consent [21,23]. We genotyped a total of 104 binary polymorphisms on the Y chromosome (M6, M49, M32, M42, M94, P85, M181, M182, M168, M294, P144, M174, M15, M179, P99, M96, P150, P147, M75, P143, M216, RPS4Y711, M8, M105, M38, M217, M356, P55, M428, P96, M201, P257, M342, P287, M69, M52, P127, P123, M258, U179, M253, M450, P215, P209, M304, M172, M9, P128, M147, P60, P79, P261, P263, M11, M20, M76, M317, M357, P256, M106, M5, M353, P118, P195, M214, M231, P191, P186, M119, P31, M122, P198, M45, P281, M242, M306, M173, P231, M124, P249, P204, P202, M70, M320, M128, P63, Tat, P203, M103, M50, M95, PK4, M111, M88, SRY465, P49, 47z, M324, M121, M164, M159, M7, M134 and M117) according to iPLEX Assay [24] using a Sequenom Mass ARRAY iPLEX Platform (Sequenom, Hamburg, Germany). To assign specific Y chromosomal haplogroup or Y lineage to each individual, we employed a phylogenetic hierarchical approach based on Y-DNA Haplogroup Tree YSOGG 2016 [25]. The use of human subjects for this study was ethically approved by Chiang Mai University, Thailand.

**Table 1:** Samples included in this study and basic indices of genetic diversity.

			HVR-I				Y-STR				Y-SNP	
Population	Code	Group	N	*h*	sd	No. of haplotypes	N	*h*	sd	No. of haplotypes	N	No. of haplogroups
Khon Muang 1	KM1	KM	50	0.967	0.0121	31	21	1	0.0147	21	21	7
Khon Muang 2	KM2	KM	41	0.974	0.0103	25	16	0.9917	0.0254	15	16	5
Khon Muang 3	KM3	KM	36	0.967	0.0141	22	15	1	0.0243	15	15	7
Khon Muang 4	KM4	KM	52	0.98	0.0085	36	29	1	0.0091	29	29	9
Khon Muang 5	KM5	KM	43	0.933	0.0222	22	20	1	0.0158	20	21	8
Khon Muang 6	KM6	KM	45	0.954	0.0193	29	22	1	0.0137	22	22	12
Khon Muang 7	KM7	KM	46	0.934	0.0201	21	23	0.9921	0.0154	21	23	6
Khon Muang 8	KM8	KM	45	0.961	0.0143	26	22	0.9913	0.0165	20	22	11
Khon Muang 9	KM9	KM	45	0.932	0.028	25	22	0.987	0.0201	20	22	7
Khon Muang 10	KM10	KM	30	0.922	0.023	12	14	0.989	0.0314	13	14	5
Mon	MO	MK	41	0.921	0.0216	16	15	0.981	0.0308	13	18	6
Lawa 1	LW1	MK	46	0.959	0.0134	25	25	0.95	0.0237	15	25	4
Lawa 2	LW2	MK	50	0.913	0.0178	15	25	0.9533	0.0296	18	25	4
Khuen	KH	TK	60	0.967	0.0096	31	29	0.9877	0.0133	25	24	7
Lue 1	LU1	TK	51	0.915	0.0274	23	25	0.99	0.0142	22	24	9
Lue 2	LU2	TK	44	0.878	0.0257	14	21	0.981	0.0197	17	22	6
Lue 3	LU3	TK	50	0.988	0.0072	39	26	0.9969	0.0117	25	26	7
Lue 4	LU4	TK	46	0.932	0.0197	19	24	0.9783	0.0205	20	19	4
Yuan 1	YU1	TK	39	0.969	0.0145	26	20	0.9895	0.0193	18	19	6
Yuan 2	YU2	TK	50	0.974	0.0094	30	25	0.9833	0.0171	21	23	7
Yuan 3	YU3	TK	50	0.966	0.0116	28	26	0.9692	0.022	20	24	11
Yuan 4	YU4	TK	44	0.948	0.0147	21	21	0.9952	0.0165	20	19	7
Yong	YO	TK	62	0.965	0.0088	31	31	0.9892	0.0108	26	26	9
Shan	SH	TK	43	0.972	0.0106	26	19	0.9942	0.0193	18	20	7

N = number of samples; *h* = haplotype diversity: sd = standard deviation. The linguistic affiliation of the populations is coded as: TK = Tai-Kadai; MK = Mon-Khmer; KM = Khon Mueang. Y-STRs and mtDNA-HVR1 were retrieved from previous studies [10,23]

In order to investigate the origin of the KM populations from both maternal and paternal perspectives using a simulations based analysis, we assembled two datasets in which we collected genetic information for 17 Y-STRs loci: DYS19, DYS388, DYS389a, DYS389b, DYS390, DYS391, DYS392, DYS393, DYS426, DYS434, DYS435, DYS436, DYS437, DYS439, DYS460, DYS461, Y-GATA-A10 and mtDNA HVR-I sequence (Table 1). A total of 536 genotypes for Y-STR and 1,109 for mtDNA-HVRI sequences were retrieved from literature [10,23].

### Statistical analyses

We employed a discriminant analysis of principal components (DAPC) [26] on both uniparental datasets to define genetic relationship among KM, MK and TK groups. The DAPC analysis can be used to investigate the relationship between populations optimizing the variation between- and within-groups while being free from assumptions about Hardy-Weinberg equilibrium or linkage equilibrium [26]. We first assessed the best number of clusters in the HVR-I and Y-STR datasets using the *find*.*clusters* function in adegenet 1.3–1 [27] and compared the results of 5 independent runs using a custom made R script. We then ran the DAPC analysis with 100,000 iterations and checked for the consistency of the groups founded.

An exploratory analysis such as the DAPC however do not account for geographic location of the samples, thus precluding the visualization of geographic locations where gene flow between populations is either hindered or facilitated. Estimated Effective Migration Surfaces (EEMS) which employed individual based migration rates can be used to visualize zones with higher/lower migration with respect to the overall rate [28]. The region under study is first divided in a grid of demes and the individuals are assigned to the deme closest to their sampling location. The matrix of effective migration rates is then computed by EEMS based on the stepping-stone model [29] and on resistance distances [30]. We applied EEMS to a matrix of pairwise *Φ*_*st*_ distances constructed on the mtDNA dataset using Arlequin 3.5 [31] and on the 17 Y-STRs using the script available from Github at https://github.com/dipetkov/eems. We averaged three runs each with 200, 300, 400 and 500 demes to produce the final EEMS surface, as the number of demes simulated during the grid construction phase can influence the scale of the deviation from overall migration detected [28]. Each single run consisted of 200,000 burn—in steps followed by 500,000 MCMC iterations sampled every 1000 steps. We plotted the averaged EEMS and checked for MCMC convergence using the *rEEMSplots* package in R v 3.2.2.

**Fig 1:**
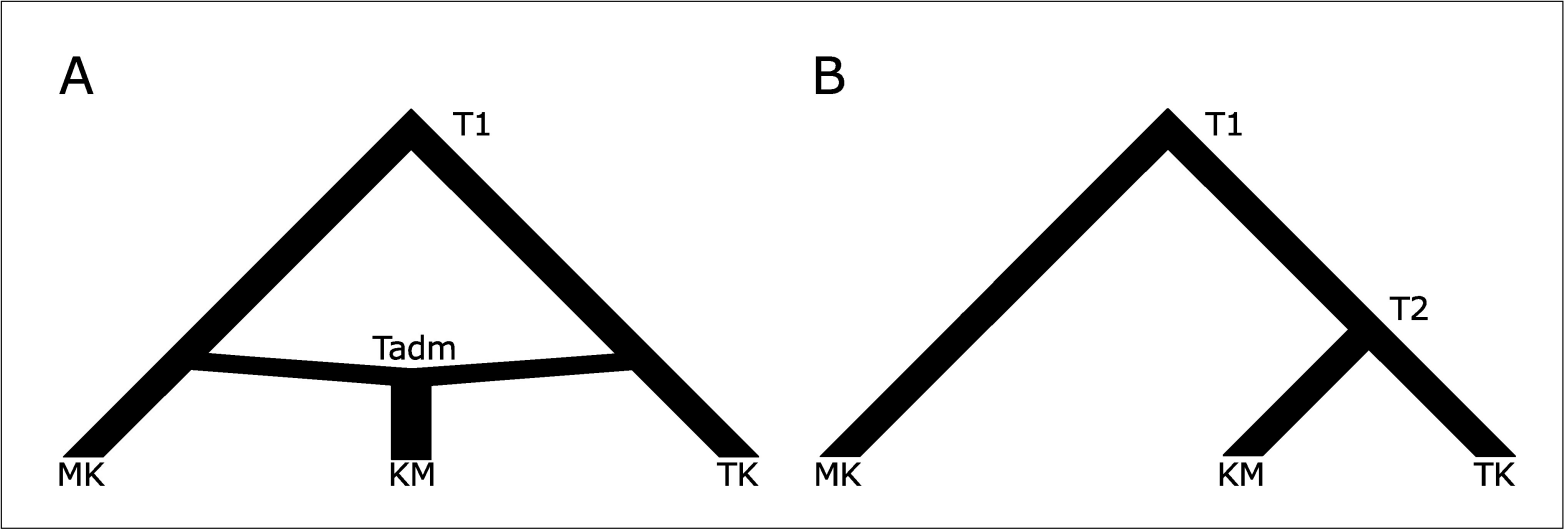
The models tested in the ABC analysis on Mon-Khmer (MK), Khon Mueang (KM) and Tai-Kadai (TK) populations: admixture (A) and tree-like (B). Times of populations split and admixture are indicated as T1, T2 and Tadm.

In order to unravel the origin of the KM population, we employed an Approximate Bayesian Computation (ABC) approach on both the HVR-I and Y-STR datasets. We first constructed two competing models (Fig 1), which are admixture and tree-like models based on our previous results [21,22].

In the admixture model, the KM population originated as a consequence of an admixture event from the parental populations, the MK and TK groups. The tree-like model postulates instead a recent separation of the KM and TK populations and a split of this combined population from the MK ones further back in time. For both the admixture and the tree-like models, we assumed constant effective population sizes based on historical records (S1 Table) and that the prior distributions were all uniform. The ABC methodology allows us to simulate thousands of genetic datasets for both of our competing models by means of the coalescent theory. These datasets are generated taking into account the prior distribution associated with each of the model parameters while being also composed of the same number of individuals and type of genetic markers that characterize the observed ones. The genetic variation in both the observed and simulated datasets is then summarized using a fixed set of statistics and compared using Euclidean distance. The posterior probabilities of each model are computed using a weighted multinomial logistic regression (LR) considering the simulations, which generate summary statistics most similar to the observed ones, as shown by smallest Euclidean distances. In the LR methodology, the model is considered as the categorically dependent variable in the simulations and the summary statistics as the predictive variables. The regression is local around the vector of observed summary statistics and the probability of each model is finally evaluated at the point corresponding to the observed vector of summary statistics. Maximum likelihood is used to estimate the *β* coefficients of the regression model. To evaluate the stability of the models’ posterior probabilities, we considered different thresholds by considering different number of retained simulations for LR (25 000, 50 000, 75 000 and 100 000 best simulations). To generate the simulated datasets, we used the software package ABCtoolbox, running 500 000 simulations for each model. To calculate the models’ posterior probabilities, we used R scripts from http://code.google.com/p/popabc/source/browse/#svn%2Ftrunk%2Fscripts, modified by SG. To summarize the genetic information contained in both the HVR-I and Y-STR datasets we calculated two arrays of statistics within and between populations. For the mitochondrial DNA dataset, we considered the number of haplotypes, the number of private polymorphic sites, Tajima’s D, the mean number of pairwise differences for each population, the mean number of pairwise differences between populations and pairwise *F*_*st*_. When we analysed Y-STR, we used as summary statistics the mean and the sd. over loci in each population of four parameters: the number of alleles, haplotype diversity, modified Garza–Williamson index and the allelic range. Finally, as the genetic heterogeneity was observed in KM populations [23], we repeated the ABC approach outlined in Fig 1 for each KM population (KM1 to KM10).

## Results

### Y haplogroups

**Fig 2:**
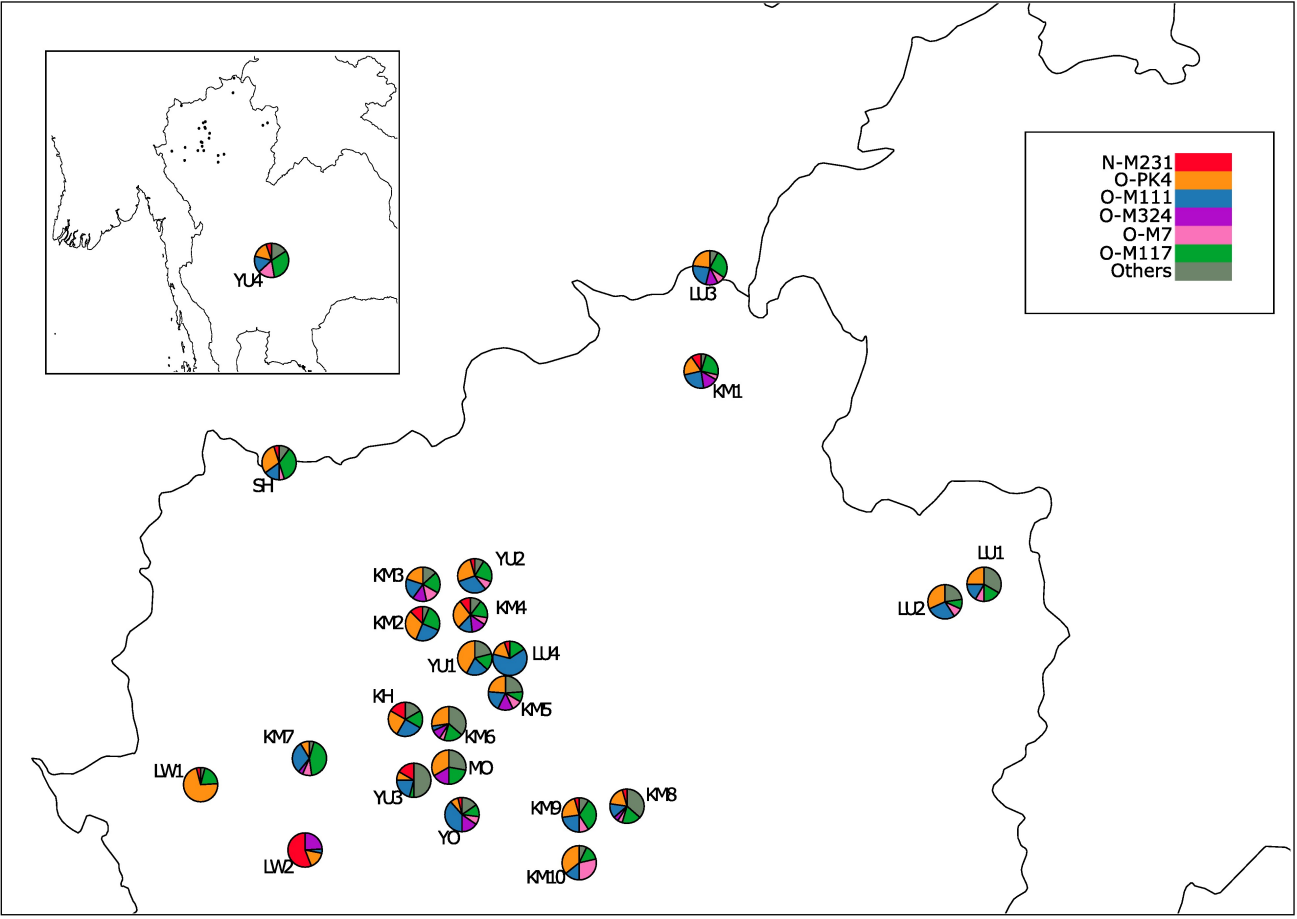
Location of the studied populations and frequencies of the 7 major haplogroups obtained in Northern Thailand.

The haplogroup frequencies in each studied population are listed in Table 2 and represented geographically in Fig 2, while the evolutionary relationships between them are presented in S1 Fig. Haplogroup O-PK4 (or O1b1a1) is the most diffuse, being present in all populations with frequencies ranging from 7.7% (YO) to 72.0% (LW1). The O-M117 (O2a2b1a1) is a haplogroup which is also commonly found (4.3% - 43.5%) in almost all the populations, except LW2. The differentiation of LW2 is also evident from the elevated frequency of haplogroup N-M231 (56.0%), which occurred only at minor frequencies in the other populations (ranging from 4.0% to 16.6%). However, LW2 is the only MK population harbouring O-M111 (O1b1a1a1a1a), a haplogroup otherwise widely distributed in both KM and TK. The similarity between KM and TK groups is further shown by the shared presence of haplogroups C-M217 (C2), D-M15 (D1a1), O-P203 (O1a1a) and O-M7 (O2a2a1a2). This similarity is enhanced when considering the populations inhabiting the central part of northern Thailand. Some other haplogroups commonly present in KM, such as C-M130 and O- M324 (O2a), seem to be shared with some MK (MO, LW2) and some TK (LU3, YO, YU3) groups. Interestingly, while being geographically removed from the other sampled locations, YU4 showed similar haplogroup distribution related to the other TK populations.

**Table 2:** Frequencies of Y chromosomal SNP haplogroups in KM, MK and TK populations.

		Haplogroups and diagnostic SNPs																						
Population	Size	C	C1b1a1	C2	CF	E1	D1a1	J2	K	H1a1	L1a1	N	NO	O1a1a	O1a2	O1b1a1	O1b1a1a1a1a	O2	O2a	O2a2a1a2	O2a2b1	O2a2b1a1	R1	R2a
		M130	M356	M217	P143	P173	M15	M172	P128	M52	M76	M231	P195	P203	M50	PK4	M111	M122	M324	M7	M134	M117	M173	P249
KM1	21											9.5		4.8		19.1	23.8		14.3	4.7		23.8		
KM2	16			6.2								12.5				31.3	25					25		
KM3	15	6.7												6.7		20	20		13.3	13.3		20		
KM4	29			3.5		3.5				3.5		10.3				27.6	13.8		13.8	6.8		17.2		
KM5	21			4.8			4.8							14.3		23.8	19		14.3	9.5		9.5		
KM6	22	4.6	4.6	4.6			4.6	4.6								27	4.6	4.6	9	4.6		18.2	9	
KM7	23						4.4									8.7	30.3		4.4	8.7		43.5		
KM8	22	4.6		9							9	4.6		4.6	9	18.2	13.6		4.6	4.6		18.2		
KM9	22						4.6		4.6			4.6				22.7	22.7			9		31.8		
KM10	14	7.1														35.7	14.3			28.6		14.3		
MO	18							5.6						5.6		33.2			16.7			22.2		16.7
LW1	25						4					4				72						20		
LW2	25											56				16	4		24					
KH	24						8.3					16.7		4.2		25	25				4.1	16.7		
LU1	24			8.3			4.2						4.2	12.4		25	16.7	4.2		8.3		16.7		
LU2	22													9.1	13.6	31.8	27.3			9.1		9.1		
LU3	26												4	4		23	23		11.5	7.7		26.8		
LU4	19											5.3				15.8	63.2					15.8		
YU1	19			5.3												42.1	21				10.5	15.8	5.3	
YU2	23			4.4								4.4				26.1	30.4	4.3		8.7		21.7		
YU3	24	4.2		12.5			4.2					16.6		4.2		8.3	20.8	16.6			4.2	4.2	4.2	
YU4	19									5.3		5.3		10.5		15.8	15.8			15.8		31.5		
YO	26						3.9					3.9		7.7	3.9	7.7	38.4		15.4	7.7		11.4		
SH	20				5							5		5		30	15			5		35		

### Population structure

**Fig 3:**
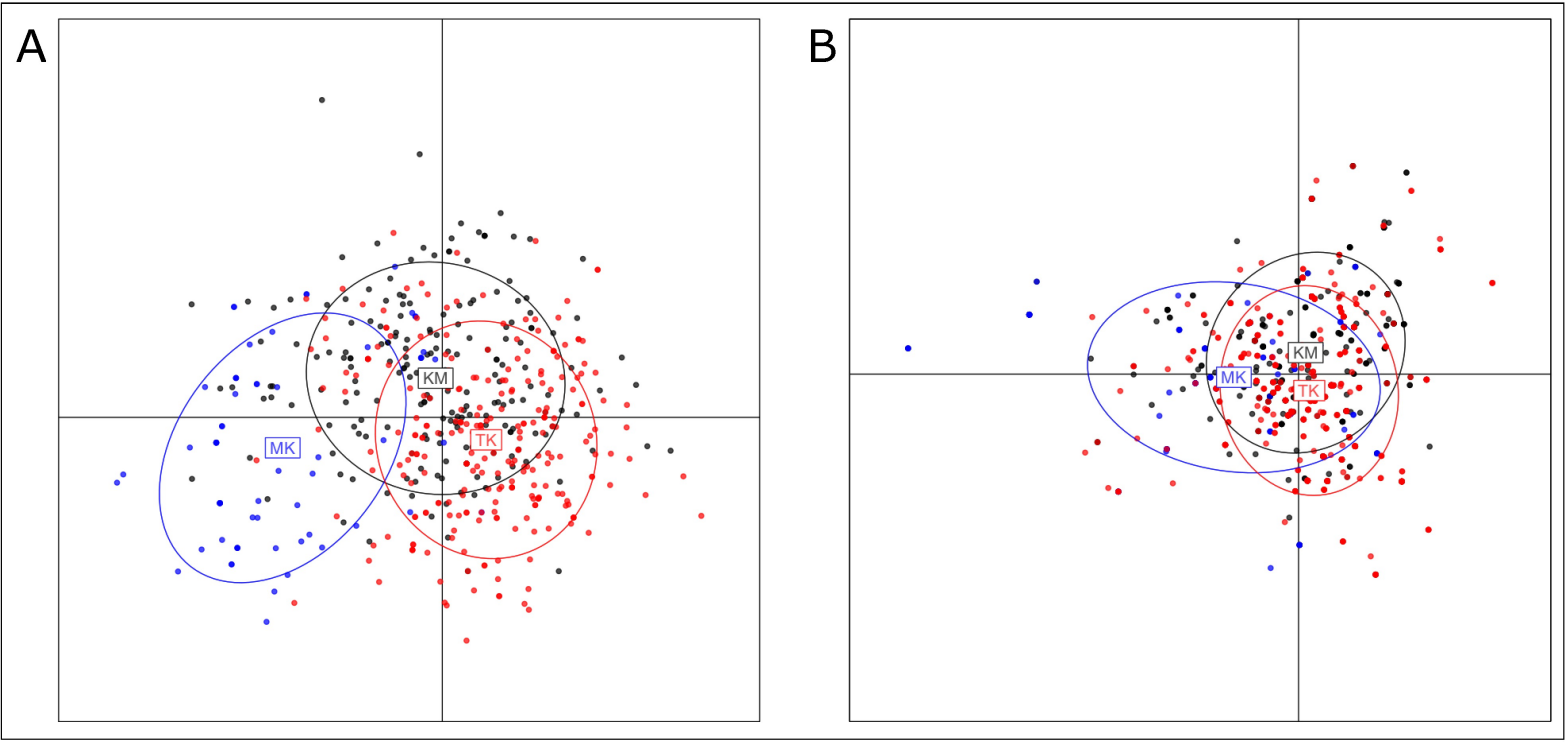
DAPC analysis on the Y-STR (A) and mtDNA-HVR1 (B) dataset. Scatterplots show three linguistic grouping: TK (red), KM (black) and KM (blue).

The DAPC analysis failed to find a clear most supported number of *K* in both uniparental datasets (S2A and S2B Fig ). However, once we assigned each sample to either its original population (S2C and S2D Fig) or language group (Fig 3A and 3B), the resulting scatterplots highlighted several patterns. The DAPC analysis based on the Y-STR dataset revealed a general overlapping of the Tai-Kadai and Khon Mueang clusters while the Mon-Khmer populations seemed clearly distinct, especially LW1 and LW2. When grouped separately, some the Khon Mueang populations inhabiting central northern Thailand such as KM3, KM4, KM5 and KM1 slightly departed from the general trend of similarity with the majority of Tai-Kadai. These populations appeared closer to the Mon-Khmer populations than the rest of KM. The Tai-Kadai population clusters were largely overlapping, with the notable exception of the SH that fell closer to LW1 and MO. The DAPC based on the mtDNA-HVR1 dataset presented less separation among both linguistic and population-based clusters, showing the overall similarity between maternal lineages in northern Thailand.

**Fig 4:**
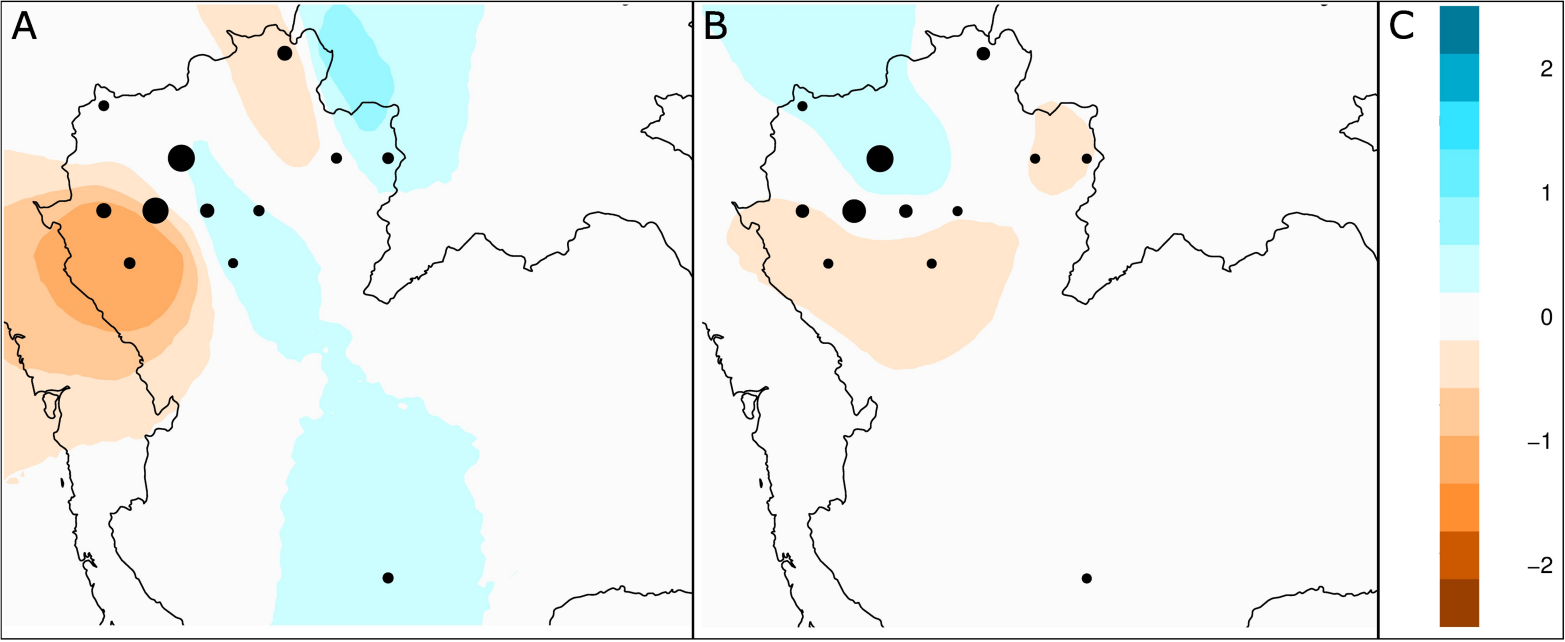
EEMS analysis of effective migration rates (*m*) on the Y-STRs (A) and mtDNA-HVR1 (B) datasets. The effective migration rate is represented on a log10 scale represented on the right. Areas showing negative values (orange) represent possible barriers to gene-flow while zones with positive values (blue) correspond to places of increased gene-flow with respect to normal IBD (white).

The EEMS surfaces showed an overall pattern of good connectivity between neighbouring populations in northern Thailand with only moderate reductions/increment of migration rates (Fig 4A and 4B). Especially evident in the Y-STR dataset, the geographic outlier population, YU4, was connected with TK and KM populations residing in the central part of northern Thailand by a corridor of high effective migration (Fig 4A). These northern Thai populations were in turn well connected with each other and with the eastern Lue (LU1 and LU2) populations. The strongest barrier in the Y-STR dataset was, not surprisingly, the one separating LW1 and LW2 populations, leading to lower migration rates with surrounding KM and TK populations (KM7, YU3 and YO). The MO did not conform to the isolation pattern presented by the Lawa, showing higher than expected migration rates with TK and KM populations. The EEMS surface based on the mtDNA-HVR1 dataset (Fig 4B) highlighted a weak spatial structure compared with the Y-STR dataset. We observed lower than expected migration rates between LW1, LW2 and the KM populations from the southern part of northern Thailand (KM8, KM9, KM10), as well as a feeble barrier between LU1 and LU2. However, higher migration rates indicate the connection between the SH and populations residing in the central part of northern Thailand.

### Model selection

The posterior probabilities from ABC analysis of the two considered evolutionary models are presented in Table 3. For the Y-STR dataset, we found that the tree-like model postulating a recent split between the ancestors of modern KM and TK populations provided a better explanation for the KM origin than an admixture model. The high and stable posterior probabilities over a different number of retained simulations confirmed the chosen tree-like model. Once we repeated the ABC analysis for each separated KM population (S2 Table), the results weakly supported tree-like model in most of the KM populations. The ABC analysis failed to support the tree-like model in KM9. The results based on mtDNA HVR-I were also less indicative of supporting the tree-like model than the admixture, however, the results obtained from simulations conducted on separated KM populations supported the tree-like model in almost all of the KM populations (e.g. KM2, KM3, KM4, KM6, KM7 and KM10). There was only one instance of KM5, in which the admixture model was preferred for the mtDNA-HVR1 dataset.

**Table 3:** Posterior probabilities of each model performed by ABC analysis under weighted multinomial logistic regression.

	HVR-I		Y-STR	
Threshold	Admixture model	Tree-like model	Admixture model	Tree-like model
**25000**	0.566	0.434	0.294	0.706
**50000**	0.479	0.521	0.297	0.703
**75000**	0.468	0.532	0.299	0.701
**100000**	0.487	0.513	0.302	0.698

## Discussion

The investigation of Y chromosomal lineages in northern Thai populations revealed that the majority of the sampled individuals could be assigned to one of three common haplogroups: O-PK4 (O1b1a1), O-M117 (O2a2b1a1) and O-M111 (O1b1a1a1a1a). These lineages are also prevalent in Chinese and other Southeast Asian populations [32–35]. The overall pattern of haplogroups distribution was also generally homogeneous in our studied populations, with subhaplogroups of O1 and O2 reaching the highest frequencies amongst the studied individuals. This was especially true for TK and KM populations. Interestingly, the MO from northern Thailand show the presence of haplogroups usually found in South, Central and West Asia: R-P249 (R2a) and J-M172 (J2) [36]. Connection between the ethnic Mon and populations from South and Central Asia was already proposed from previous identification of mtDNA lineage W3a1b [20]. Both groups of the MK speaking LW groups showed high differences between each other and from other populations, presenting low levels of haplogroup diversity (Table 1) with high frequencies of O-PK4 (O1b1a1) (72% in LW1) and N-M231 (56% in LW2). Haplogroup N-M231 is prevalent in today’s TK and Hmong-Mien speaking populations, as well as in Han of southern China [37]. We also detected haplogroup C-M130 and its sublineages (C-M356 and C-M217) in almost all KM and YU populations, as well as in one Lue group (LU1). Haplogroup C-M130 has been found mainly in Mongolia and in Korea, while in Southeast Asia it reaches high frequencies in the eastern part of Indonesia. C-M217 is, instead, typically present at high frequencies across northeast Asia [38]. It is worth to note that we observed haplogroup D1-M15 in several TK and KM populations, although at low frequencies. High frequency of this lineage was reported in China especially in Tibet, Quiang and Yao [39]. To account for the presence of C and D lineages in our TK groups, we speculate that paternal admixture among several ethnolinguistic groups in the area of southern China were heavily influenced by Han and Mongol expansion from the north [40–43]. This would have happened before the southward migration of the TK ancestor to northern Thailand, which could contribute to the presence of C and D lineages in these populations.

In agreement with earlier studies of mtDNA and autosomal STRs variation [20–22] that reported genetic differentiation of MK speaking groups in northern Thailand, the two LW groups appeared to differ from other populations and from each other, as indicated by the DAPC results (S2C Fig). A lower level of gene flow caused by the presence of geographic barriers may be the driven factor of this differentiation, as suggested by the EEMS surfaces (Fig 4). On the other hand, the Mon is less genetically differentiated from the KM and TK groups. Although the genetic origin of the Mon is related to South Asia, recent admixture with the Tai sources could have been the main force that shaped the genetic variation of the northern Thai Mon.

To investigate the origin of the KM, we proposed two demographic models, which summarized previous hypothesis on their origin. The first scenario depicted an admixture event involving local MK populations and migrating TK groups, while a second tree-like model suggested that the KM originated following a recent split from the ancestors of modern TK populations (Fig 1). We then proceeded to test these hypotheses with ABC simulations on both maternally and paternally inherited data. When all the KM individuals were pooled together, the most supported model was the one postulating a close relationship between these populations and TK (Table 3). This demographic pattern was clearer when Y-STRs were employed instead of mtDNA, possibly suggesting significant maternal contribution from local MK speaking on current KM populations. This conclusion was reinforced when the single KM populations were considered separately. In some specific cases (KM5, S2 Table), the admixture scenario obtained higher posterior probabilities than the tree-like one, highlighting the importance of considering small scale local processes when investigating the history of a population. In conclusion, we observe contrasting pattern of paternal and maternal genetic variation with a clearer genetic structure in the Y-STRs than in the mtDNA-HVR1 sequences. Our different approaches suggest nonetheless a common origin between KM and TK populations, previously proposed to be located in southern China [22]. After an initial migration southward, fragmentation process in separate villages and contact with local MK speaking people could have promoted secondary events of admixture, especially in the matrilineal lineage (S2 Table). Although some models and populations compared are different from our previous mtDNA genome study [22], the results are confirmed with the additional inclusion of post-settlement contacts with MK populations. Future work employing complete Y sequences and/or in depth autosomal information from northern Thai and surrounding populations will be crucial to determine the migration origin and to evaluate the full impact of secondary admixture.

## Supporting information

S1 Fig Evolutionary tree for the 23 Y-chromosome haplogroups obtained for northern Thai populations. The names of the lineages are reported on the right while the markers that define them are shown along the branches of the tree. Lineage names with an asterisk refer to internal nodes of the tree.
(PDF)

S2 Fig BIC resulting from averaging 5 runs of *find*.*clusters* on both Y-STR and HVR-I datasets: points represents the mean of the iterations and bars the standard deviation (A, B). Scatterplots of the DAPC conducted on the Y-STR and HVR-I datasets when individual sequences where grouped based on populations (C, D).
(PDF)

S1 Table Prior distributions of model parameters. Labels are detailed in Fig 2; Time (T1, T2 and Tadm) are expressed in generations. (DOCX)

S2 Table Posterior probabilities of each model performed by ABC analysis on single KM populations. (DOCX)

S3 Table Newly genotyped individuals and haplogroups assignment. (XLS)
