# Supplementary figures and images for: Y chromosomal evidence on the origin of northern Thai people
Source: PLoS One. 2017 Jul 24;12(7):e0181935. doi: 10.1371/journal.pone.0181935 (PMC5524406; doi:10.1371/journal.pone.0181935)

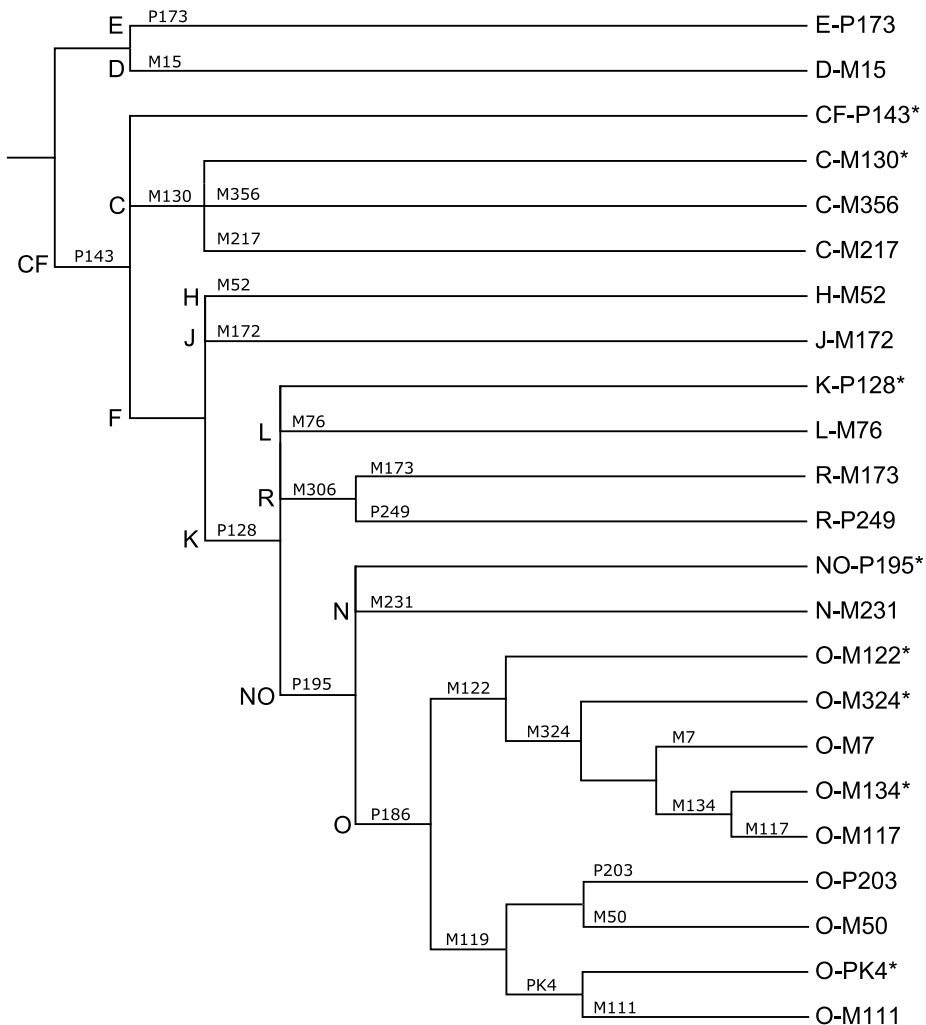

Supplement: S1 Fig — The names of the lineages are reported on the right while the markers that define them are shown along the branches of the tree. Lineage names with an asterisk refer to internal nodes of the tree. (PDF) [file pone.0181935.s001.pdf]

**A**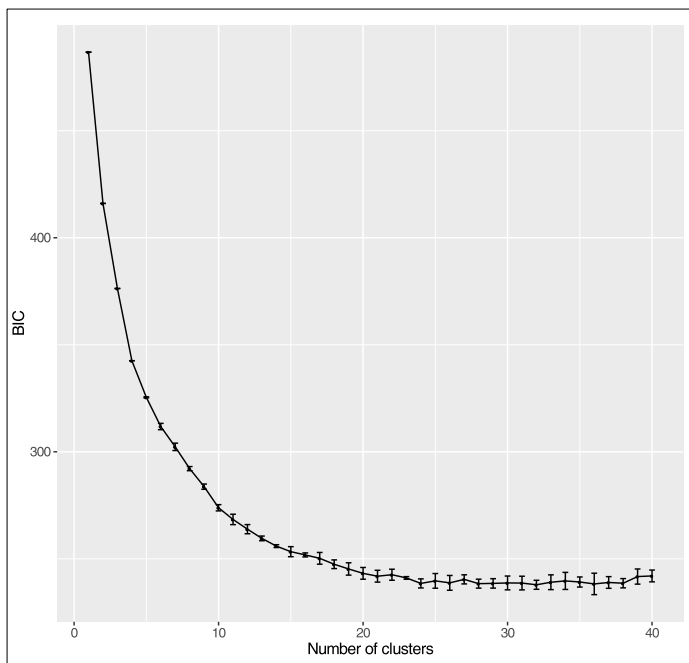**B**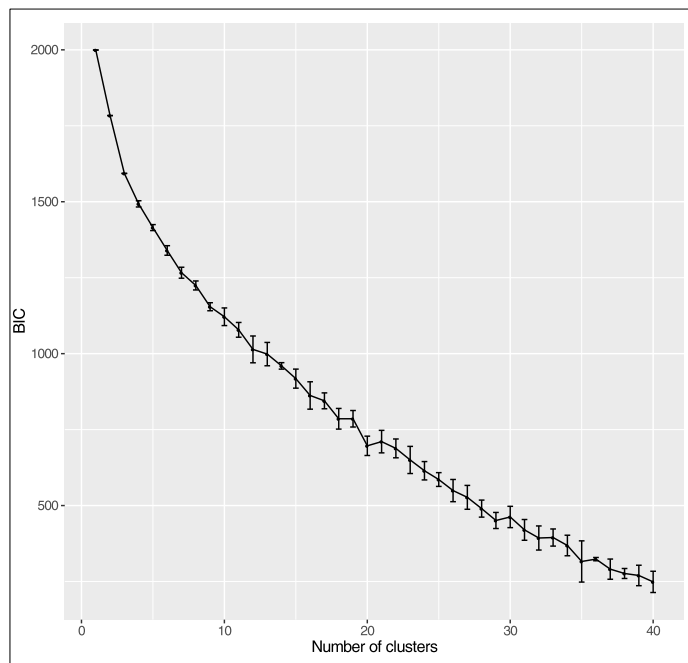**C**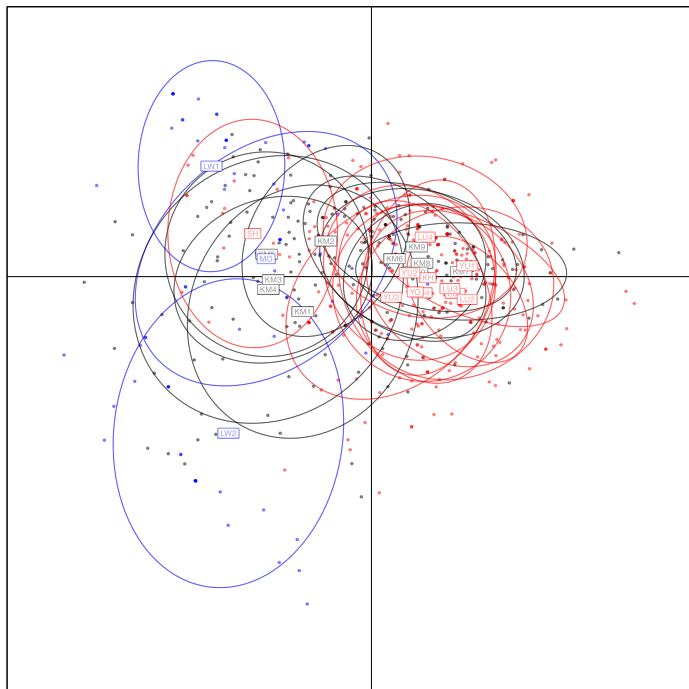**D**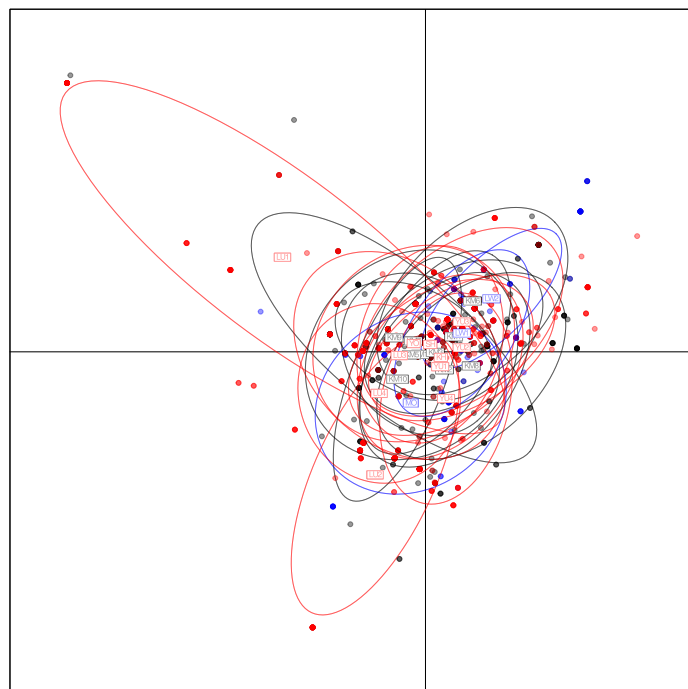

Supplement: S2 Fig — BIC resulting from averaging 5 runs of find.clusters on both Y-STR and HVR-I datasets: points represents the mean of the iterations and bars the standard deviation (A, B). Scatterplots of the DAPC conducted on the Y-STR and HVR-I datasets when individual sequences where grouped based on populations (C, D). (PDF) [file pone.0181935.s002.pdf]
