# Supplementary material for: Y chromosomal evidence on the origin of northern Thai people
Source: PLoS One. 2017 Jul 24;12(7):e0181935. doi: 10.1371/journal.pone.0181935 (PMC5524406; doi:10.1371/journal.pone.0181935)
Supplement: S1 Table — (DOCX) [file pone.0181935.s003.docx]

| **Admixture Model** | | |
| --- | --- | --- |
| **Ne MK** | uniform | 1,000 - 100,000 |
| **Ne KM** | uniform | 10,000 - 1,000,000 |
| **Ne TK** | uniform | 10,000 - 1,000,000 |
| **T1** | uniform | 100 - 200 |
| **Tadm** | uniform | 30 - 50 |
|  |  |  |
| **Tree-like model** | | |
| **Ne MK** | uniform | 1,000 - 100,000 |
| **Ne KM** | uniform | 10,000 - 1,000,000 |
| **Ne TK** | uniform | 10,000 - 1,000,000 |
| **T1** | uniform | 100 - 200 |
| **T2** | uniform | 30 - 80 |

**HVR-I mutation rate =** 1.6 x 10^-7^ [1]

**Y-STR mutation rate** = 8.7 x 10^-5^ [2]

**References**

1. Soares P, Ermini L, Thomson N, Mormina M, Rito T, Röhl A, et al. Correcting for Purifying Selection: An Improved Human Mitochondrial Molecular Clock. Am J Hum Genet. 2009 Jun;84(6):740-59.
2. Willems T, Gymrek M, Poznik GD, Tyler-Smith C; 1000 Genomes Project Chromosome Y Group., Erlich Y. Population-Scale Sequencing Data Enable Precise Estimates of Y-STR Mutation Rates. Am J Hum Genet. 2016 May 5;98(5):919-33
