# Supplementary material for: Y chromosomal evidence on the origin of northern Thai people
Source: PLoS One. 2017 Jul 24;12(7):e0181935. doi: 10.1371/journal.pone.0181935 (PMC5524406; doi:10.1371/journal.pone.0181935)
Supplement: S2 Table — (DOCX) [file pone.0181935.s004.docx]

|  | **ALL KM** | | | |  |  | **KM1** | | | |  |  | **KM2** | | | |
| --- | --- | --- | --- | --- | --- | --- | --- | --- | --- | --- | --- | --- | --- | --- | --- | --- |
|  | **HVR-I** | | **Y-STR** | |  |  | **HVR-I** | | **Y-STR** | |  |  | **HVR-I** | | **Y-STR** | |
| Threshold | Admixture | Tree-like | Admixture | Tree-like |  | Threshold | Admixture | Tree-like | Admixture | Tree-like |  | Threshold | Admixture | Tree-like | Admixture | Tree-like |
| **25000** | 0.566 | 0.434 | 0.294 | 0.706 |  | **25000** | 0.495 | 0.505 | 0.494 | 0.506 |  | **25000** | 0.312 | 0.688 | 0.513 | 0.487 |
| **50000** | 0.479 | 0.521 | 0.297 | 0.703 |  | **50000** | 0.472 | 0.528 | 0.452 | 0.548 |  | **50000** | 0.291 | 0.709 | 0.459 | 0.541 |
| **75000** | 0.468 | 0.532 | 0.299 | 0.701 |  | **75000** | 0.445 | 0.555 | 0.449 | 0.551 |  | **75000** | 0.280 | 0.720 | 0.448 | 0.552 |
| **100000** | 0.487 | 0.513 | 0.302 | 0.698 |  | **100000** | 0.427 | 0.573 | 0.445 | 0.555 |  | **100000** | 0.275 | 0.725 | 0.449 | 0.551 |
|  | **KM3** | | | |  |  | **KM4** | | | |  |  | **KM5** | | | |
|  | **HVR-I** | | **Y-STR** | |  |  | **HVR-I** | | **Y-STR** | |  |  | **HVR-I** | | **Y-STR** | |
| Threshold | Admixture | Tree-like | Admixture | Tree-like |  | Threshold | Admixture | Tree-like | Admixture | Tree-like |  | Threshold | Admixture | Tree-like | Admixture | Tree-like |
| **25000** | 0.430 | 0.570 | 0.520 | 0.480 |  | **25000** | 0.309 | 0.691 | 0.482 | 0.518 |  | **25000** | 0.607 | 0.393 | 0.487 | 0.513 |
| **50000** | 0.392 | 0.608 | 0.468 | 0.532 |  | **50000** | 0.303 | 0.697 | 0.445 | 0.555 |  | **50000** | 0.559 | 0.441 | 0.446 | 0.554 |
| **75000** | 0.378 | 0.622 | 0.455 | 0.545 |  | **75000** | 0.293 | 0.707 | 0.445 | 0.555 |  | **75000** | 0.547 | 0.453 | 0.430 | 0.570 |
| **100000** | 0.366 | 0.634 | 0.451 | 0.549 |  | **100000** | 0.290 | 0.710 | 0.446 | 0.554 |  | **100000** | 0.538 | 0.462 | 0.435 | 0.565 |
|  | **KM6** | | | |  |  | **KM7** | | | |  |  | **KM8** | | | |
|  | **HVR-I** | | **Y-STR** | |  |  | **HVR-I** | | **Y-STR** | |  |  | **HVR-I** | | **Y-STR** | |
| Threshold | Admixture | Tree-like | Admixture | Tree-like |  | Threshold | Admixture | Tree-like | Admixture | Tree-like |  | Threshold | Admixture | Tree-like | Admixture | Tree-like |
| **25000** | 0.417 | 0.583 | 0.459 | 0.541 |  | **25000** | 0.399 | 0.601 | 0.460 | 0.540 |  | **25000** | 0.486 | 0.514 | 0.533 | 0.467 |
| **50000** | 0.383 | 0.617 | 0.420 | 0.580 |  | **50000** | 0.374 | 0.626 | 0.401 | 0.599 |  | **50000** | 0.447 | 0.553 | 0.482 | 0.518 |
| **75000** | 0.367 | 0.633 | 0.413 | 0.587 |  | **75000** | 0.358 | 0.642 | 0.404 | 0.596 |  | **75000** | 0.437 | 0.563 | 0.479 | 0.521 |
| **100000** | 0.357 | 0.643 | 0.423 | 0.577 |  | **100000** | 0.343 | 0.657 | 0.414 | 0.586 |  | **100000** | 0.424 | 0.576 | 0.476 | 0.524 |
|  | **KM9** | | | |  |  | **KM10** | | | |  |  |  |  |  |  |
|  | **HVR-I** | | **Y-STR** | |  |  | **HVR-I** | | **Y-STR** | |  |  |  |  |  |  |
| Threshold | Admixture | Tree-like | Admixture | Tree-like |  | Threshold | Admixture | Tree-like | Admixture | Tree-like |  |  |  |  |  |  |
| **25000** | 0.517 | 0.483 | 0.501 | 0.499 |  | **25000** | 0.372 | 0.628 | 0.531 | 0.469 |  |  |  |  |  |  |
| **50000** | 0.483 | 0.517 | 0.504 | 0.496 |  | **50000** | 0.342 | 0.658 | 0.493 | 0.507 |  |  |  |  |  |  |
| **75000** | 0.469 | 0.531 | 0.506 | 0.494 |  | **75000** | 0.324 | 0.676 | 0.475 | 0.525 |  |  |  |  |  |  |
| **100000** | 0.454 | 0.546 | 0.508 | 0.492 |  | **100000** | 0.307 | 0.693 | 0.475 | 0.525 |  |  |  |  |  |  |
